# Supplementary figures and images for: A signal capture and proofreading mechanism for the KDEL-receptor explains selectivity and dynamic range in ER retrieval
Source: eLife. 2021 Jun 17;10:e68380. doi: 10.7554/eLife.68380 (PMC8248988; doi:10.7554/eLife.68380)

Figure 1 Supplement 1d. Source data

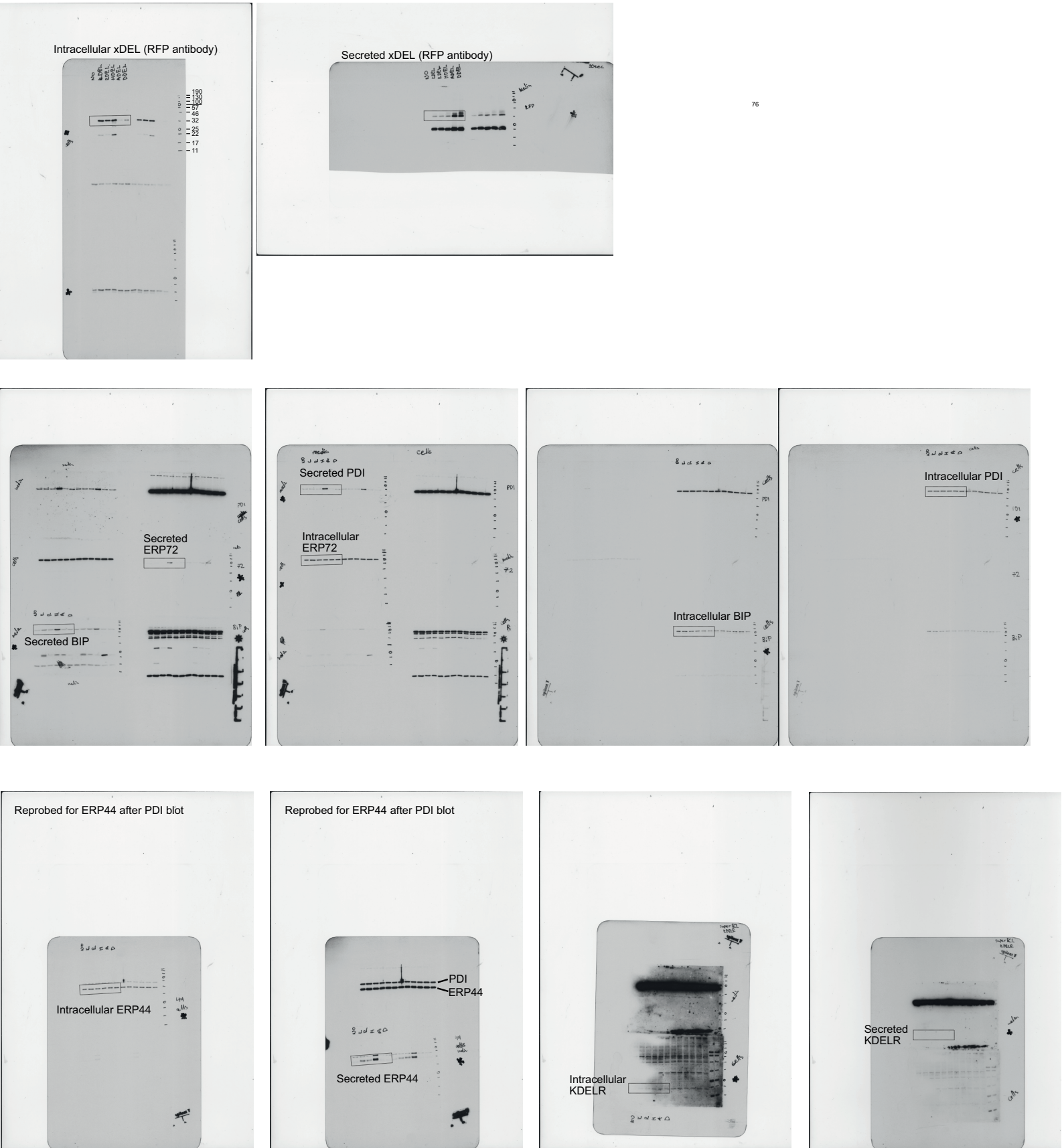

Supplement: Figure 1—figure supplement 1—source data 2. — Individual blot files are provided as a ZIP archive. [file elife-68380-fig1-figsupp1-data2.pdf]
