# Supplementary material for: A signal capture and proofreading mechanism for the KDEL-receptor explains selectivity and dynamic range in ER retrieval
Source: eLife. 2021 Jun 17;10:e68380. doi: 10.7554/eLife.68380 (PMC8248988; doi:10.7554/eLife.68380)
Supplement: Supplementary file 1. — Values in parentheses are for the highest resolution shell. [file elife-68380-supp1.docx]

**Table S1. Crystallographic Data Collection Statistics.** *Values in parentheses are for the highest resolution shell.*
