# Supplementary material for: A signal capture and proofreading mechanism for the KDEL-receptor explains selectivity and dynamic range in ER retrieval
Source: eLife. 2021 Jun 17;10:e68380. doi: 10.7554/eLife.68380 (PMC8248988; doi:10.7554/eLife.68380)
Supplement: Supplementary file 2. — The free energy difference is expressed in kcal/mol and the sum is the ensemble free energy difference between KDEL and HDEL. HID signifies that the histidine is protonated at δ nitrogen and HIE means that the histidine is protonated at ε nitrogen, while HIP means that both positions are protonated. The occupancy of protonation state is computed at pH seven and expressed as a percentage (%). [file elife-68380-supp2.docx]

The free energy difference is expressed in kcal/mol and the sum is the ensemble free energy difference between KDEL and HDEL. HID signifies that the histidine is protonated at δ nitrogen and HIE means that the histidine is protonated at ε nitrogen, while HIP means that both positions are protonated. The occupancy of protonation state is computed at pH 7 and expressed as a percentage (%).

| **Table S2.** Free energy differences for HDEL protonation states and KDEL | | | |
| --- | --- | --- | --- |
|  | WT | W120A | W120F |
| (kcal/mol) | Free energy difference of replacing KDEL with HDEL | | |
| HDEL (HID) | 1.6±1.2 | -0.5±0.6 | -1.1±0.8 |
| HDEL (HIE) | 2.4±0.6 | 0.4±0.7 | 0.6±1.3 |
| HDEL (HIP) | -1.9±0.2 | -0.8±0.3 | -0.9±0.2 |
| Sum | -1.8±1.4 | -0.3±0.9 | -0.7±1.6 |
|  | Occupancy of each protonation state | | |
| **HDEL (HID)** | 0.4 | 35.6 | 54.0 |
| **HDEL (HIE)** | 0.1 | 8.3 | 3.8 |
| **HDEL (HIP)** | 99.5 | 56.1 | 42.3 |
