# Supplementary material for: A signal capture and proofreading mechanism for the KDEL-receptor explains selectivity and dynamic range in ER retrieval
Source: eLife. 2021 Jun 17;10:e68380. doi: 10.7554/eLife.68380 (PMC8248988; doi:10.7554/eLife.68380)
Supplement: Supplementary file 3. — The cation-π interaction is the energy resulting from induction calculated at the level of sSAPT0/jun-cc-pVDZ. The π-π interaction is the sum of exchange and correlation energy. The sum is the sum of cation-π and π-π energy. [file elife-68380-supp3.docx]

The cation-π interaction is the energy resulting from induction calculated at the level of sSAPT0/jun-cc-pVDZ. The π-π interaction is the sum of exchange and correlation energy. The sum is the sum of cation-π and π-π energy.

| **Table S3.** Cation-π and π-π contributions between receptor W120 and the -4 histidine in the retrieval signal | | | | |
| --- | --- | --- | --- | --- |
| (kcal/mol) | Protonation state | cation-π | π-π | Sum |
| WT | HID | -0.7 | -3.4 | -4.1 |
|  | HIE | -0.7 | -3.3 | -4.0 |
|  | HIP | -3.4 | -3.2 | -6.6 |
| W120A | HID | -0.1 | -0.5 | -0.5 |
|  | HIE | -0.1 | -0.5 | -0.5 |
|  | HIP | -0.8 | -0.4 | -1.2 |
| W120F | HID | -0.6 | -2.0 | -2.6 |
|  | HIE | -0.5 | -1.7 | -2.2 |
|  | HIP | -2.8 | -1.6 | -4.4 |
